# Case Report: Irreversible Watery Diarrhea, Severe Metabolic Acidosis, Hypokalemia and Achloridria Syndrome Related to Vasoactive Intestinal Peptide Secreting Malignant Pheochromocytoma

**DOI:** 10.3389/fendo.2021.652045

**Published:** 2021-03-17

**Authors:** Aurelio Negro, Ignazio Verzicco, Stefano Tedeschi, Nicoletta Campanini, Magda Zanelli, Emanuele Negri, Enrico Farnetti, Davide Nicoli, Barbara Palladini, Rosaria Santi, Davide Cunzi, Anna Calvi, Pietro Coghi, Luigi Gerra, Riccardo Volpi, Gallia Graiani, Aderville Cabassi

**Affiliations:** ^1^ Internal Medicine and Secondary Hypertension Center, Ospedale Sant’Anna di Castelnovo Ne’ Monti, Azienda Unità sanitaria Locale – IRCCS di Reggio Emilia, Reggio Emilia, Italy; ^2^ Centro Ipertensione Arteriosa e Studio Malattie Cardiorenali, S.S. Fisiopatologia Medica, Clinica Medica Generale e Terapia Medica, Parma, Italy; ^3^ Pathology Unit, Department of Medicine and Surgery, University Hospital of Parma, Parma, Italy; ^4^ Pathology Unit, Ospedale Sant’Anna di Castelnovo Ne’ Monti, Azienda Unità sanitaria Locale – IRCCS di Reggio Emilia, Reggio Emilia, Italy; ^5^ High Care Internal Medicine Unit, Ospedale Sant’Anna di Castelnovo Ne’ Monti, Azienda Unità sanitaria Locale – IRCCS di Reggio Emilia, Reggio Emilia, Italy; ^6^ Molecular Biology Laboratory, Ospedale Sant’Anna di Castelnovo Ne’ Monti, Azienda Unità sanitaria Locale – IRCCS di Reggio Emilia, Reggio Emilia, Italy; ^7^ Histology and Histopathology Unit, Dental School, University of Parma, Parma, Italy

**Keywords:** pheochromocytoma, watery diarrhea hypokalemia achlorhydria syndrome, arterial hypotension, vasointestinal peptide (VIP), metabolic acidosis

## Abstract

**Background:**

Pheochromocytoma (PHEO) clinical manifestations generally mirror excessive catecholamines secretion; rarely the clinical picture may reflect secretion of other hormones. Watery diarrhea, hypokalemia and achlorhydria (WDHA) is a rare syndrome related to excessive secretion of vasoactive intestinal peptide (VIP).

**Clinical Case:**

A 73-year-old hypotensive man affected by adrenal PHEO presented with weight loss and watery diarrhea associated with hypokalemia, hyperchloremic metabolic acidosis (anion gap 15 mmol/l) and a negative urinary anion gap. Abdominal computed tomography scan showed a right adrenal PHEO, 8.1 cm in maximum diameter, with tracer uptake on ^68^GaDOTA-octreotate positron emission tomography. Metastasis in lumbar region and lung were present. Both chromogranin A and VIP levels were high (more than10 times the normal value) with slightly elevated urine normetanephrine and metanephrine excretion. Right adrenalectomy was performed and a somatostatin analogue therapy with lanreotide started. Immunostaining showed chromogranin A and VIP co-expression, with weak somatostatin-receptor-2A positivity. In two months, patient clinical conditions deteriorated with severe WDHA and multiple liver and lung metastasis. Metabolic acidosis and hypokalemia worsened, leading to hemodynamic shock and exitus.

**Conclusions:**

A rare case of WDHA syndrome caused by malignant VIP-secreting PHEO was diagnosed. High levels of circulating VIP were responsible of the rapidly evolving clinical picture with massive dehydration and weight loss along with severe hyperchloremic metabolic acidosis and hypokalemia due to the profuse untreatable diarrhea. The rescue treatment with lanreotide was unsuccessful because of the paucity of somatostatin-receptor-2A on VIP-secreting PHEO chromaffin cells.

## Introduction

Pheochromocytomas (PHEO) and sympathetic paragangliomas are rare neuroendocrine tumors arising from chromaffin cells in the medulla of the adrenal glands or from neural crest-derived ganglia. PHEO are usually functional and secrete catecholamines (1). They may manifest with an array of clinical symptoms including headaches, sweating, palpitations, paroxysmal or persistent hypertension, and various signs or symptoms related to catecholamines secretion or, in rare cases, to isolated or combined release of other hormones such as somatostatin, renin, adrenocorticotropic hormone, parathyroid hormone, erythropoietin, enkephalins, calcitonin, neuropeptide Y (2–4). Watery diarrhea associated with hypokalemia and achlorhydria (WDHA) characterizes a rare syndrome first described by Verner and Morrison in 1958 (5) linked to hypersecretion of vasoactive intestinal peptide (VIP). Such a clinical situation was also termed pancreatic cholera because of severe diarrhea resembling those related to Vibrio cholera infection. VIP is a 28-amino acids peptide, member of the secretin/glucagon/pituitary adenylyl cyclase-activating peptide hormone superfamily. VIP is able to regulate gastric acid secretion, intestinal contractility and anion secretion, exocrine pancreas release, vasodilation by acting on two G-protein-coupled receptors VPAC1 and VPAC2 (6). Enteric anion secretion depends from VPAC1 receptor-mediated adenylyl cyclase and protein kinase A activation leading to cystic fibrosis transmembrane conductance regulator stimulation, responsible for the secretion of both Cl^-^ and HCO_3_^-^ in the ileum and colon (7–9). WDHA associates with severe metabolic acidosis due bicarbonate wasting but also with abnormal glucose tolerance due to VIP-mediated glycogenolytic effect on the liver, with hypercalcemia and tetany-related hypomagnesemia due to diarrhea. The patients also experience facial flushing because of VIP vasodilating effect. VIP-secreting tumors usually originate in the pancreas or much more rarely along the sympathetic chain or in the gut and the skin (10, 11). Here, we report an emblematic case of intractable and refractory WDHA syndrome in a patient with a VIP-secreting malignant adrenal PHEO.

## Case Report

**Figure 1:**
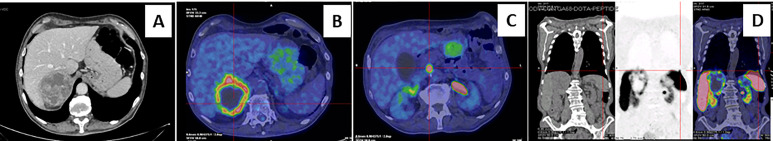
Abdominal computed tomography scan and Positron emission tomography imaging. Contrast-enhanced abdominal CT scan showed inhomogeneous right adrenal mass measuring 8.1 x 7.7 x 7.9 cm **(A)**. A 18F-fluorodeoxyglucose PET-CT showed an area of high uptake in the right adrenal gland, with a prevailing peripheral signal and central hypoactivity, and in the lumbar region suspected of adenopathic localization **(B, C)**. A 68GaDOTA-octreotate (DOTATATE) PET confirmed previous localizations and also detected a high uptake are at the base of the left lung **(D)**.

**Figure 2:**
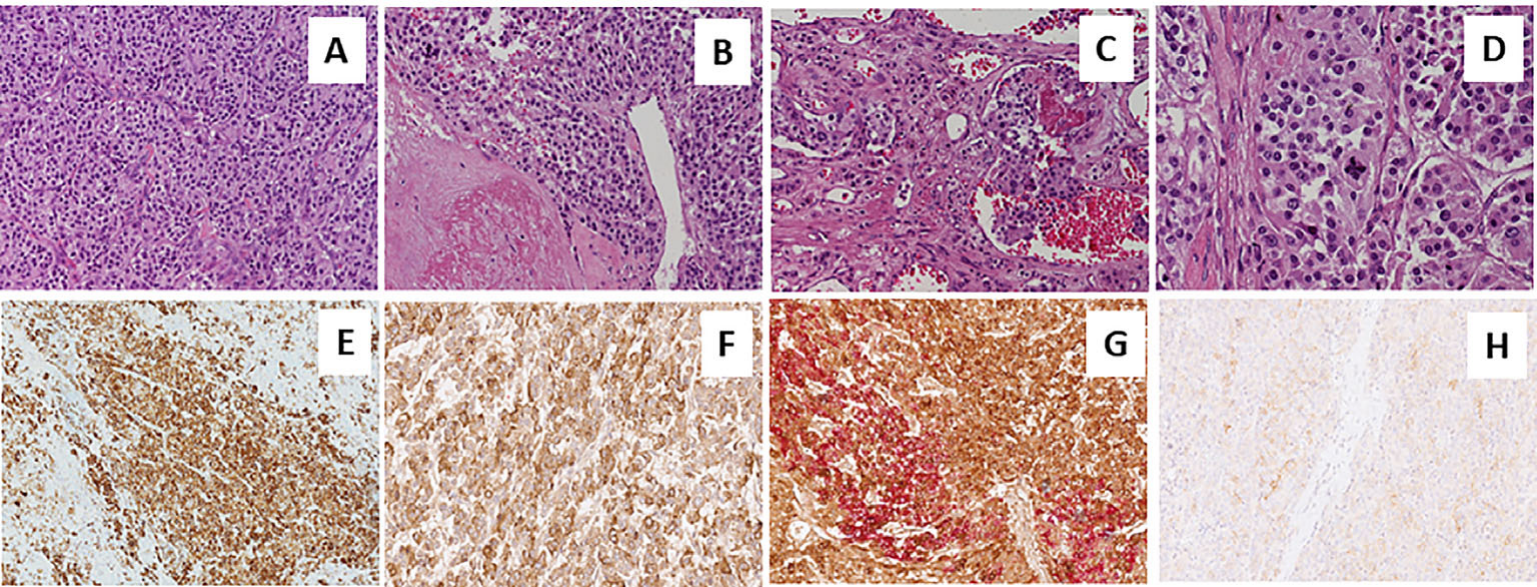
Sections from right adrenal gland Morphologic analysis with hematoxylin-eosin staining in **(A–D)** at different magnification. Panels show a pheochromocytoma, with a typical zellballen pattern of growth (**A**, 20X), with perivascular cell cuffing around a blood vessel called pseudo-rosette pattern (**B**, 20X), with neoplastic cells inside vascular spaces (**C**, 20X), and at greater magnification (**D**, 40X), an atypical mitotic figure. Positive immunohistochemical staining of Chromogranin A in Panel **(E)**, VIP **(F)** and anti-Somatostatin Receptor 2A (SSTR2A, **H**). Double immunostaining showing the co-expression of Chromogranin A (brown) and VIP (red) **(G)**.

**Figure 3:**
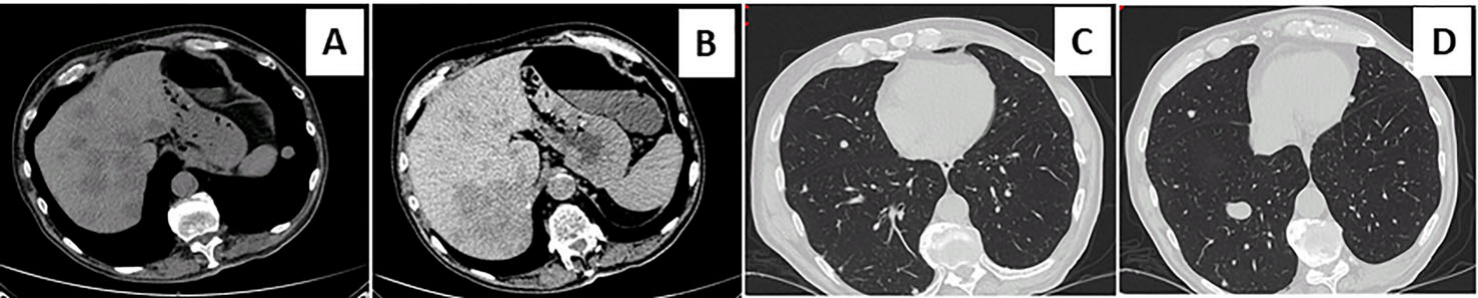
Metastatic diffusion at chest and hepatic computed tomography scan. Contrast-enhanced abdominal and chest computed tomography scan showed the appearance of multiple metastatic pulmonary and hepatic nodules **(A–D)**.

A 73-year-old Caucasian man was referred to our hospital for evaluation of a right PHEO, diagnosed two months before at another hospital, after the identification of a large retroperitoneal mass on abdominal computed tomography (CT). At that time, the patient experienced abdominal discomfort, unintentional weight loss of approximately 5 Kg within the previous 3 months, associated to sporadic episodes of watery diarrhea. At admission to our hospital, the patient was moderately dehydrated and tachypnoic. He denied any history of headache, palpitations, sweating, or hypertension. He reported episodes of watery diarrhea, up to 5-6 times a day and 2-3 times a week, without blood or mucus. He also had no relevant familial history of endocrine nor cancer diseases but only a paternal history of arterial hypertension. Physical examination showed blood pressure (BP) of 100/67 mmHg and heart rate of 88 beats/min; no significant orthostatic pressure gradient was measured. BP values, evaluated on several occasions, were 94/58 and 91/62 mm Hg. Laboratory tests showed a hypokalemia (3.3 mmol/L) with metabolic acidosis (pH 7.29, HCO3^-^ 19 mmol/L), a serum magnesium level of 1.5 mg/dl and fasting blood glucose of 149 mg/dl. A 24-h urinary sample showed only a slight increase in normetanephrine excretion, 638 μg (normal values: 162-528/day), while metanephrine and methoxytyramine resulted within normal range. Serum chromogranin A was elevated (1028 ng/ml, normal values 20-100), as well as neuron-specific enolase level (NSE 35.7 nl/ml, normal values 1.0-13.5). Plasma cortisol, adrenocorticotropic hormone, thyroid-stimulating hormone, thyroxine, parathyroid hormone, and calcitonin were within the normal ranges. Contrast enhanced abdominal CT scan confirmed the presence of inhomogeneous right adrenal mass measuring 8.1 x 7.7 x 7.9 cm (**Figure 1A**). A ^18^F-fluorodeoxyglucose (18F-FDG) positron emission tomography (PET) coupled with CT showed an area of high uptake (maximum standardized uptake value, SUV max 8.6) in the right adrenal gland, with a prevailing peripheral signal and central hypoactivity, and another area of high uptake (SUV max 9.6) in the lumbar region suspicious of lymph node localization (**Figures 1B, C**). In addition, ^68^GaDOTA-octreotate (DOTATATE) PET confirmed the peripheral high uptake (SUV max 6) in the right adrenal gland and the high uptake area (SUV max 3.7) in the lumbar region; a high uptake (SUV max 4.5) was also detected at the base of the left lung (**Figure 1D**). Based on these results, patient diagnosis was metastatic adrenal PHEO. Intravenous fluid infusion, sodium bicarbonate, potassium aspartate, magnesium sulphate supplementations were started allowing an improvement of clinical condition and blood pressure levels. Then, after 10-days pre-operative treatment with low dose alpha1-adrenergic antagonist doxazosin (given just before bed), he underwent surgical resection of the tumor. The patient had an uneventful postoperative course, except for sporadic watery diarrhea. Gross examination revealed a 10x8x6 cm brownish-yellow, friable adrenal mass. Histology showed a highly cellular tumor made up of monotonous medium-sized cells with discrete nuclear pleomorphism and mild hyperchromasia. Mitotic figures were above 3/10 high power fields, with some atypical mitoses. The cells were arranged in nests with areas of diffuse growth in more than 10% of the tumor. Confluent areas of necrosis were present. Foci of capsular and vascular invasion were noted as well as extension into periadrenal adipose tissue. The histological features were consistent with a malignant PHEO, with a PASS score (Pheochromocytoma of the Adrenal Gland Scaled Score) of 20 (**Figures 2A–D**), indicating a high risk of aggressive cellular behavior (PASS≥4). DNA genetic analysis of the patient with a next generation sequencing (NGS) approach using Trusight One Sequencing Panel by Illumina, revealed a synonymous single nucleotide variant of gene SDHA [rs6555055, NM_004168.2:c.619A>C, (p.Arg207=)] indicated by ClinVar database as associated to “probably benign” catecholamine-secreting PHEO (12). The patient was discharged in satisfactory clinical condition. Therapy with lanreotide, a somatostatin analogue, at a dose of 60 mg once a month was initiated. At 2 months, multiple metastatic pulmonary and hepatic nodules were identified on CT scan (**Figures 3A–D**). The patient once again experienced abdominal discomfort, 4 kg weight loss, yet only sporadic watery diarrhea. Peptide receptor radionuclide therapy and sunitinib, a multi-targeted receptor tyrosine kinase inhibitor, were scheduled. In the meantime, lanreotide therapy was increased to 120 mg once a month. However, after about one month, the patient was re-admitted with a 10-day history of severe watery diarrhea, up to 20 times in 24 hrs, accompanied by nausea, vomiting and occasionally quick flushing. At presentation, he was suffering and markedly dehydrated. Physical examination showed BP of 90/67 mmHg, heart rate of 120 beats/min, the pulse was fast and weak, the breath was fast and short, the skin cold and clammy, and the urination was decreased. Laboratory tests were as follows: blood urea nitrogen 96 mg/dl; serum creatinine 3.5 mg/dl; Na^+^ 136 mmol/l; K^+^ 2.5 mmol/l; Cl^-^ 115 mmol/l; pH 7.08; HCO_3_^-^ 5.5 mmol/l; Pa CO2 30 mm Hg; Pa O2 67 mm Hg; lactate 5 mmol/l (normal values 0.5-2.2); serum anion gap 15 mmol (corrected for serum albumin levels 16 mmol); urine anion gap was negative. Serum prealbumin was 29 mg/dl (normal values 15-35) and albumin 3.9 g/dl (normal values 3.5-5.0). At that time, serum chromogranin A was 2896 ng/ml and neuron-specific enolase 49.6 ng/ml. Twenty-four hours urinary normetanephrine excretion was 920.4 μg, while metanephrine resulted at 432.6 μg (normal values 64-302 μg/day). VIP plasma levels were measured, and circulating values were more than 10 times the upper normal limit (1285 pg/ml, normal values 18-100). Cardiac ultrasound showed a reduced left ventricular ejection fraction (35%). Due to the emerging clinical picture, histological sections were re-evaluated with additional immunostainings. Sections were stained with the following primary antibodies: anti-Chromogranin A (clone LK2H10 ready to use; Ventana-Roche), Anti-Vasoactive intestinal polypeptide -VIP (rabbit 1:500; Biogenex) and anti-Somatostatin Receptor 2A -SSTR2A (rabbit 1:100; Bio-Trend). The sections were immunostained with HRP Polymer (Optiview DAB IHC Detection kit; Roche) in accordance with the manufacturer’s specifications. Negative controls consisted of substituting normal mouse serum for the primary antibodies. A set of sections adjacent to these used for single labelling with VIP, was used for double labelling with Chromogranin A. The second antibody was immunostained with AP Polymer (Ultraview Universal Alcaline Phosphatase Red Detection Kit; Roche). Permanent red chromogen was used for staining development. Immunostaining revealed strong positivity for neuroendocrine marker chromogranin A and VIP (**Figures 2E, F**); a large number of cells co-expressed chromogranin A and VIP (**Figure 2G**). Weak was the positivity for SSTR2A (**Figure 2H**). A diagnosis of VIP-secreting PHEO was rendered. The patient was then transferred to the intensive care unit. He was managed with intensive intravenous fluid hydration, potassium salts and bicarbonates, as well as with octreotide (0.1 mg/8 h s.c.), sunitinib 50 mg/day and loperamide. However, his diarrhea worsened with further exacerbation of metabolic acidosis (pH 6.99, HCO_3_^-^ 4.3 mmol/l), leading to hemodynamic instability and shock. He died five days later.

## Discussion

Our patient represents a new case of WDHA syndrome caused by a malignant VIP-secreting PHEO with massive dehydration, severe hyperchloremic metabolic acidosis and hypokalemia due to the profuse diarrhea. VIP-secreting PHEO is an extremely rare tumor and from the first description given in 1975 by Lowry et al, only about twenty five cases of PHEO associated with WDHA syndrome have been reported, so far (13–20).

As a general rule, the clinical manifestations of PHEO are very challenging to interpret, reflecting the prevailing hormonal secretion by chromaffin cells, generally represented by catecholamines. In the present case, the modest increase in normetanephrine and metanephrine levels did cause the classic picture of PHEO with arterial paroxysmal or persistent hypertension, headaches, palpitations, anxiety, and diaphoresis. As the main secreted hormone was VIP, diarrhea and arterial hypotension were the main clinical manifestations. VIP elicits a potent vasodilation not only of the splanchnic circulation (gastric, small intestine and colon), but even systemically through a direct effect on VPAC2 receptor of vascular smooth muscle and through VPAC1 receptor-mediated nitric oxide release from endothelial cells (21). VIP systemic vasodilating effect, largely exceeding the vasoconstrictor effect of catecholamines, associated to diarrhea-related volume depletion contributes to the hypotension observed in our patient (14, 16). Episodic facial flushing reported by our patient could be the result of VIP direct arterial skin vasodilatory effect.

Watery diarrhea, hypokalemia, metabolic acidosis due of enteric bicarbonate loss and hypo- or rarely achlorhydria are clinical features of VIP-secreting tumors and generally begin several years before diagnosis. Our patient reported sporadic diarrhea and body weight loss just 3 months before, followed by a rapid clinical worsening. The accelerated time course of WDHA syndrome is a feature of VIP-secreting PHEO compared to pancreatic islet cell tumor, bronchogenic carcinomas, and medullary thyroid carcinomas secreting VIP (14, 16, 17). The clinical condition is clearly dependent from VIP effect on intestinal secretion and motility and inhibition of gastric acid secretion through gastrin release reduction. In a physiologic situation, VIP, derived from the subsequent cleavage and metabolic steps of 170-amino-acid precursor prepro-VIP, is released from several population of enteric nerve terminals, both cholinergic and non-cholinergic, from myenteric and submucosal nerve plexi of gastrointestinal tract, from cholinergic nerves in the pancreatic islet, and from enteric lymphoid tissues (6, 22). Following enterocyte VIP-mediated VPAC1 receptor activation, HCO3^-^, Cl^-^ and water secretion is stimulated in the ileum and colon but mainly in the duodenum by the apical Cl^-^/HCO_3_^–^ exchanger and the cystic fibrosis transmembrane conductance regulator (9). Pancreatic secretion of HCO3^-^ is also increased by VIP (23). In our case the autonomous and non-regulated release of massive amount of VIP by the malignant and metastatic PHEO led to refractory and fulminant diarrhea with severe fluid, potassium and bicarbonate depletion (7–9, 24). A severe hyperchloremic metabolic acidosis with a serum anion gap of 16 mmol and a negative urine anion gap [(Na^+^+K^+^)-Cl^-^] was observed in our case. To avoid a wrong interpretation of the acid-base disorder it is important to remember that clinicians still refer to a normal range of the serum gap anion [Na^+^- (Cl^-^+ HCO3^-^)] between 9 and 16 mmol/l but this is no longer true because of the change of measuring electrolyte techniques that brought normal values ​​to lower ranges (4-11 mmol/l) (25). A 16 mmol/l of serum anion gap is just slightly above the normal range and this is an important issue because such a disturbance along with the negative urine anion gap would indicate that kidney is still able to excrete ammonium, and the cause of metabolic acidosis is mainly ascribed to the severe loss of bicarbonates related to VIP-mediated secretory diarrhea. However, while this is the most plausible explanation, we must consider some other factors that can interfere with the acid-base disorder in our case. The patient had an acute reduction in renal function from excessive volume depletion and arterial hypotension. Two conditions have to be taken into account; first, the acute renal functional reduction, that can interfere and reduce the significance of the urinary gap as an indicator of ammonium ion secretion. Second, the arterial hypotension can increase the serum anion gap by the generation of serum lactate, despite being modest the rise observed in our patient (26). Furthermore, a high generation of serum lactate could also be linked to excessive cell proliferation by the VIP-secreting malignant PHEO (27). Therefore, a mixed acidosis both with normal and high serum anion gap can better describe the acid-base disturbance of our patient as also evidenced by the excessive reduction of plasma bicarbonates compared to the slight increase in the serum anion gap. Severe metabolic acidosis determines hemodynamic and inflammatory consequences impairing cardiac contractile function and inducing arterial hyporeactivity through nitrogen and oxygen reactive species generation, altered catecholamine and intracellular calcium signaling (28–32). The altered glucose tolerance of our patient is also related to the effect of VIP high circulating levels, stimulating glucagon release and activating liver glycogenolysis, and to acidosis-mediated insulin resistance development, although a contribution of the modest catecholamines excess cannot be excluded (33, 34).

Based on the concept that neuroendocrine tumors widely express somatostatin receptors and as recommended by Food and Drug Administration and reported in literature (35, 36), we treated our patient with lanreotide acetate, a synthetic cyclical octapeptide analog of somatostatin to inhibit VIP release and improve the WDHA syndrome. Octreotide has been reported to inhibit VIP release from pancreatic tumors reducing diarrhea in 75% of cases (37) and in a case of WDHA syndrome caused by a VIP-producing PHEO to be able to reverse a shock condition (14). The inefficacy of lanreotide treatment in our case was probably due to the paucity of SSTR2A on VIP-secreting PHEO cells observed at immunohistochemistry (**Figure 2H**), as previously reported (19). The combined treatment with sunitinib, a multi-targeted receptor tyrosine kinase inhibitor, reported to be efficacious by Lebowitz-Amit et al. in terms of rapid and complete clinical response, did not show any favorable effects (17). Tumor progression and metastatic expansion in the liver and lung with massive release of VIP, unresponsive to therapies, precipitated the WDHA syndrome and patient clinical condition until his death.

In conclusion, our case showed a rare case of WDHA syndrome due to metastatic VIP-secreting PHEO, with an aggressive clinical behavior and high PASS score. Surgery did not completely eradicate the cancer and the scarcity of SSTR2A expression on tumor cells made the treatment with lanreotide unsuccessful. In order to promptly face clinical symptoms and tumor progression, careful attention should be paid to correctly interpret any non-classic symptoms of PHEO, producing and releasing in rare cases classes of hormones other than catecholamines.

## Data Availability Statement

The raw data supporting the conclusions of this article will be made available by the authors, without undue reservation.

## Ethics Statement

The studies involving human participants were reviewed and approved by Comitato Etico Unico per la Provincia di Parma. The patients/participants provided their written informed consent to participate in this study.

## Author Contributions 

Attended the patient and performed diagnostic and laboratory analysis (AN, IV, NC, MZ, EN, EF, BP, RS, DC, GG, ACb). Collected the data and contributed to the analysis of literature data (IV, ST, BP, ACa, PC, LG, ACb). Discussion of the results (AN, IV, ST, PC, RV, ACb). Wrote the paper (AN, IV, ST, ACb). All authors contributed to the article and approved the submitted version.

## Conflict of Interest

The authors declare that the research was conducted in the absence of any commercial or financial relationships that could be construed as a potential conflict of interest.
